# Supplementary material for: The impacts of COVID-19 and its policy response on access and utilization of maternal and child health services in Tanzania: A mixed methods study
Source: PLOS Glob Public Health. 2023 May 12;3(5):e0001549. doi: 10.1371/journal.pgph.0001549 (PMC10180638; doi:10.1371/journal.pgph.0001549)
Supplement: Attachment — Submitted filename: Response to reviewers_2303.docx [file pgph.0001549.s003.docx]

# Journal Requirements

1. Please send a completed 'Competing Interests' statement, including any COIs declared by your co-authors. If you have no competing interests to declare, please state "The authors have declared that no competing interests exist". Otherwise please declare all competing interests beginning with the statement "I have read the journal's policy and the authors of this manuscript have the following competing interests:"

Our response: We declare there are no COIs. We have now stated this in the ‘Additional Information’ section of the submission system.

2. Please amend your detailed Financial Disclosure statement. This is published with the article. It must therefore be completed in full sentences and contain the exact wording you wish to be published.

Our response: We have now amended the Financial Disclosure statement and added the names of the researchers who received financial support, and that the funders had no role in the study.

3. Please provide separate figure files in .tif or .eps format only and remove any figures embedded in your manuscript file. Please also ensure that all files are under our size limit of 10MB.

Our response: All the figures have now been uploaded as .tif files in the submission system (see Fig. 1-5).

4. We noticed that you used "unpublished" in the manuscript. We do not allow these references, as the PLOS data access policy requires that all data be either published with the manuscript or made available in a publicly accessible database. Please amend the supplementary material to include the referenced data or remove the references.

Our response: Unpublished references have now been removed.

5. In the online submission form, you indicated that "The anonymised interviews transcripts will be deposited on Flowrepository, and will be available upon request". All PLOS journals now require all data underlying the findings described in their manuscript to be freely available to other researchers, either 1. In a public repository, 2. Within the manuscript itself, or 3. Uploaded as supplementary information.

Our response: We have now uploaded the complete anonymised interviews database as Supplementary Information 1.

# Reviewer #1:

Dear Authors,
Thank you for the opportunity to review your manuscript. Please find some suggestions below to improve the quality of the material in your work

Abstract
Lines 49-50: You mention that you follow standards in reporting qualitative research - what standards did you follow? Just report the specific standards that was used directly.

Our response: We used the SRQR, as indicated in the Methods, and in Reference 16. We have now mentioned this in the Abstract (pag.3, ln.50).

Lines 60-63: Despite policy response suggests it was not the policy response but fear and diversion of resources and your suggestion for the future is on policy response! This doesn’t seem logical. If fear and disruption caused the issues that you discuss, then something needs to be done about that don’t you think?

Our response: We are not entirely sure what is the point the reviewer is making here. However, we recognise that our original sentence was less-than-straightforward. We have now amended it and tried to clarify that although many commentators accused Megafuli’s policies of stopping people from seeking healthcare during the pandemic, our findings suggest it was rather the fear of the pandemic itself and diversion of resources to cause the larger damage (Abstract, Ln. 60-62).

Methodology

151- There is no random sampling in qualitative research. What sampling frame was used please provide details?

Our response: In each district across the three regions, one ward was selected for community interviews and FGDs. In each ward, stratification was done to represent wealthier, middle, and poor households; thus 20 households were selected to represent three categories in each ward. Households were then selected randomly based on a sampling frame designed to achieve a balanced mix of household types. A typology of households was developed for this purpose, with each household having at least one member of reproductive age. We have now clarified the principles of this sampling frame in pag.6, Ln. 150-156.

Table 1 not clear
Your description of the details of data collection should match the table – please modify the table to ensure this

Our response: We conducted 34 in-depth interviews with policy-makers and community leaders, 11 FGD (in which 59 people participated), and 60 semi-structured interviews with households. We also examined 10 sets of health service utilisation records. We have now made Table 1 and the text in sections 2.2.2 and 2.2.3 more consistent (pag.6-8, from Ln.158 onwards).

Sampling
Lines 137-138: Why did you choose these specific locations (Unguja Mijni and Chake Chake districts?). Can you please give some details to explain your choice of the districts

Our response: We selected the three districts to cover the breadth of urban and rural settings across Tanzania mainland and Zanzibar islands – these regions have separate governments. We have now clarified this in in Ln113-118 in pag.5.

Lines 156-…: How did you go about the numbers that you report- did you observe for saturation? Please discuss this and provide details about how you detected saturation of data and how this guided your sample size.

Our response: We recruited informants to match our initial sampling framework, and stopped recruiting extra interviewees when we noticed that themes and categories started repeating in the interviews, and no new topic was emerging. We have now clarified how we identified ‘saturation point’ in pag.6, Ln. 157-160.

Data analysis
Lines 191-193: If multiple investigators went through the transcript for coding, them how did you achieve inter-coder reliability? Was this double blinded coding or otherwise? How did you triangulate. What sort of triangulation approach did you use?

Our response: Multiple coders went through the transcripts, and then one senior member of the data collection team (either ES, LM, or BTM) double-checked to ensure consistency. We used (a) method triangulation, (b) investigator triangulation, (c) data source triangulation to validate the findings. We have now clarified this in section 2.3 Data analysis (ln. 193-207).

Ethics
Lines 207-208: It is not clear to me whether you followed a two-stage consent process with an initial oral consent followed by obtaining written consent? If this is the case then you need to make it clear if the written consent was about after data collection and when exactly.

Our response: Yes, our interviewees were read upfront the study objectives and their rights to withdraw, and asked authorisation to record. Then they were asked at the end of the interviews to sign their informed consent to use the information they provided during the interviews. We have now clarified this process in Section 2.4 Ethical Considerations.

Line 209: How did you maintain confidentiality anonymity and privacy? In the methodology section of a peer reviewed publication, it is important that the readers know how you did something (that you state you did).

Our response: We anonymised the transcripts by deleting names and references to specific actors. A code comprising the location of the interview and a sequential number of the person interviewed was adopted to identify specific respondents and interviews without compromising anonymity. We have now clarified this in Section 2.4, Ln.211-219.

Results

Lines 461-473: 3.3.5 Your title says increased complications and unwanted pregnancies.
But there is no data discussed on unwanted pregnancies. Change sub-heading to reflect results presented only.

Our response: We thank the reviewer for spotting this inconsistency from previous drafts. We have now changed the title of section 3.3.5 to: ‘ Reduction of serviced for expecting mothers’ (ln. 471).

Discussion
You need to define what is linear and non-linear policy responses in the introduction so authors know the difference.

Our response: By “non-linear” we mean the govt’s ambiguous position on COVID-19 and its inconsistent policy response. We have now clarified this at the end of the Introduction (pag.5, Ln 102-105).

General observation
Requires a lot of work. I would urge the authors to focus the discussion on what is relevant to your paper. Currently, I am afraid the discussion has a lot of generic material on the topic. Shorten the initial part of the discussion. If you want to set the context globally and nationally in detail, you can do that in the Introduction. In the discussion just briefly introduce any global contexts that are relevant to the topic of the paper and discuss the relevance of each of your findings within the context of the body of relevant literature.

Our response: We have now streamlined the Discussion to make the following four key points more explicitly, and ground them in the evidence we uncovered, namely: (1) how our findings in part explain the govt’s less than straightforward policy response; (2) the evidence of indirect health harm brough by the pandemic in Tanzania; (3) our explanation for the apparent contradictions between our quantitative and qualitative data. The final paragraph present the limitations of our work (Discussion, ln.490-567).

# Reviewer #2:

Thank you for the opportunity to review this manuscript. It is well written and I have a few minor comments
- Will the health facility records be available in the data repository?

Our response: Yes, we have now made the facility records available as Supplementary Information 2.

Methods:
- Data collection, health facility records are included under qualitative methods. It seems that these records are quantitative in nature. it would be helpful to total the number of IDIs, FGDs, and SSIs conducted. In table 1, consider adding a column to total each of the different types of information.

Our response: Thank you for the useful suggestion. We have now developed Table 1 to include such column and clarify the nature of the data collected.

The data sources are skewed towrard district and community level data, which affects the generalizability of the data.

Our response: Yes, this is true. We have now added a sentence in our Limitations to qualify the generalisability of our evidence (Discussion, Ln 562-564).

Results:
- this was primarily a qualitative study. Where are the quantitative results?

Our response: This study adopted a case-study approach drawing from both qualitative and quantitative data. These latter are presented in Figure 1-5 in the Results section. Such findings are also triangulated with the qualitative evidence though the section.

Discussion/Conclusion:
- it seems there are conclusions drawn without the backing of data. For example, a "non-linear policy response," "lower uptake of antenatal, postnatal, family planning, immunization services, fewer institutional deliveries."

Our response: This point was also picked up by the other reviewer. To address these comments, we have now redrawn the Discussion to ground our reflections in the evidence from our study (see Discussion, Ln.490-560). We have also explained in the Introduction what we meant by “non-linear policy response” (see the Abstract, Ln 51; or Discussion, Ln492-495). As for the lower uptake of health services, out Fig.1-5 do show an inflection for the first year of the pandemic, although we do highlight the rebound in utilisation for the following year, and the fact that the interviewees’ opinion are only in part supported by the quantitative data on services (Discussion, Ln.497-500).
