# Supplementary material for: The impacts of COVID-19 and its policy response on access and utilization of maternal and child health services in Tanzania: A mixed methods study
Source: PLOS Glob Public Health. 2023 May 12;3(5):e0001549. doi: 10.1371/journal.pgph.0001549 (PMC10180638; doi:10.1371/journal.pgph.0001549)
Supplement: Attachment — Submitted filename: Response to editor_0304.docx [file pgph.0001549.s004.docx]

**Editor’s comments**

We appreciate the revisions made in response to the prior round of peer review, and feel that the revised draft is much stronger and more cohesive as a result. However, the terms "linear policymaking" and "nonlinear policymaking" remain unclear. Please either define these terms explicitly or remove them from the revised draft altogether.

Our response: We have now removed the word ‘linear’ and substitute through the manuscript with ‘inconsistent’ (see Abstract, ln.53; Introduction, Ln 104, Discussion Ln 492, and Conclusion, Ln 575).

1. We have noticed that you have a list of Supporting Information legends in your manuscript. However, there are no corresponding files uploaded to the submission. Please upload them as separate files with the item type 'Supporting Information'.

Our response: We have now introduced a list of supporting information files at the end of the manuscript (pag.21, Ln 290-292).

2. Please review your reference list to ensure that it is complete and correct. If you have cited papers that have been retracted, please include the rationale for doing so in the manuscript text, or remove these references and replace them with relevant current references. Any changes to the reference list should be mentioned in the rebuttal letter that accompanies your revised manuscript. If you need to cite a retracted article, indicate the article’s retracted status in the References list and also include a citation and full reference for the retraction notice.

Our response: We have now revised our reference lists, and eliminated 2 repetitions (the original ref. 15-21-25 Mfinanga et al 2021)

Reviewers' comments:

Our response: We have not found reviewer’s comments to respond to – perhaps these were not attached?
